# Feasibility of implementing a text-based symptom-monitoring program of endometrial, ovarian, and breast cancer patients during treatment

**DOI:** 10.1007/s11136-020-02660-w

**Published:** 2020-10-14

**Authors:** Michelle J. Naughton, Ritu Salani, Juan Peng, Maryam Lustberg, Cecilia DeGraffinreid, Jennifer Moon, Hibaq Loyan, Chloe M. Beverly Hery, Electra D. Paskett

**Affiliations:** 1 Division of Cancer Prevention and Control, College of Medicine, The Ohio State University, 1590 N. High St, Suite 525, Columbus, OH 43201 USA; 2 Division of Gynecologic Oncology, School of Medicine, University of California Los Angeles, Los Angeles, CA USA; 3 Department of Biomedical Informatics, College of Medicine, The Ohio State University, Columbus, OH USA; 4 Division of Medical Oncology, College of Medicine, The Ohio State University, Columbus, OH USA

**Keywords:** Symptom assessment, Text-based monitoring, Smartphones, Breast cancer, Ovarian cancer, Endometrial cancer

## Abstract

**Purpose:**

To evaluate the feasibility of implementing systematic patient symptom monitoring during treatment using a smartphone.

**Methods:**

Endometrial [*n* = 50], ovarian [*n* = 70] and breast [*n* = 193] cancer patients participated in text-based symptom reporting for up to 12 months. In order to promote equity, patients without a smartphone were provided with a device, with the phone charges paid by program funds. Each month, patients completed the Patient Health Questionnaire (PHQ-9), and 4 single items assessing fatigue, sleep quality, pain, and global quality of life during the past 7 days rated on a 0 (low) –10 (high) scale. Patients’ responses were captured using REDCap, with oncologists receiving monthly feedback. Lay navigators provided assistance to patients with non-medical needs.

**Results:**

Patients utilizing this voluntary program had an overall mean age of 60.5 (range 26–87), and 85% were non-Hispanic white. iPhones were provided to 42 patients, and navigation services were used by 69 patients. Average adherence with monthly surveys ranged between 75–77%, with breast patients having lower adherence after 5 months. The most commonly reported symptoms across cancer types were moderate levels (scores of 4–7) of fatigue and sleep disturbance. At 6 months, 71–77% of all patients believed the surveys were useful to them and their health care team.

**Conclusions:**

We established the feasibility of initiating and managing patients in a monthly text-based symptom-monitoring program. The provision of smartphones and patient navigation were unique and vital components of this program.

## Introduction

Symptom management utilizing patient-reported outcomes is an important area of focus in cancer care [1]. Cancer patients often experience symptoms related to their treatment regimens [2] and/or the disease itself [3], as well as psychosocial concerns [4, 5]. Common cancer-related symptoms, such as pain, fatigue, and depression [3, 6], may resolve after treatment completion or may persist. Past research indicates that health care providers systematically underestimate their patients’ moderate or severe symptoms compared to what patients report themselves [7]. Under-estimation, which tends to be more common than the over-estimation of patients’ symptoms [7], leads to poorer health outcomes and the under-treatment of patients [8, 9].

Routine monitoring of patients’ physical and psychological symptoms is becoming increasingly more common [5]. The use of patient-reported outcome (PRO) measures during cancer treatment has been shown to improve patients’ survival rates and result in fewer emergency department (ED) visits and hospitalizations, and less symptom burden during hospital stays [10, 11]. When patients have their symptoms addressed or at least relayed to their physicians, it has been found to improve patient satisfaction with care [12], and can help reduce patient anxiety and promote self-care [13]. In addition, monitoring patients’ health-related quality of life has been found to improve communication between patients and their health care providers [12, 14, 15].

While there are many ways to track patients’ symptoms, such as in-clinic assessments and telephone calls between visits, technological advances are making it easier to collect symptoms and adverse events using electronic devices, such as computers, tablets, or mobile phones. The use of these devices can result in faster relay times of patient information to providers [16, 17], more accurate detection of adverse events [18], and may assist in reducing the use of avoidable services like ED visits or hospitalizations [19].

Recent work has shown that health systems and providers are increasingly likely to adopt the use of electronic patient-reported outcomes (PROs) with their patients [18, 20, 21]. Successful remote assessments of PROs using electronic devices (ePROs) have used email or text-message reminders with direct links to patient forms [20], invitations through patient communication portals (e.g., MyChart) [18], and REDCap with automated emails [22, 23]. However, routine cancer care has been slower to adopt ePROs. Challenges to implementing ePROs in clinical practice have been identified, with attempts to address these barriers by involving stakeholders (physicians/staff and patients) [19, 24], and interviewing patients, caregivers, and providers to ensure the relevance of measures selected for PRO assessments [20, 25, 26].

In 2014, a systematic review was conducted on ePRO use in clinical oncology settings (*n* = 27) [27]. Results indicated that 30% of the ePRO systems reviewed were accessible from the home, and 37% were accessible from both the home and the clinic. Many of the assessments were conducted on computers or tablets, but few used cell phones. Most of the ePROs were designed to be completed by patients during active treatment (63%), but others were also used for follow-up care (40%). Reminders to complete the ePRO surveys were sent in 63% of the systems, with email being the most common method (53%), and 33% using phone, text, or letter reminders. Real-time alerts were used in 85% of systems to send patients’ responses to their providers. The systems reviewed collected a variety of PRO data that were reported to providers (e.g., current scores, longitudinal changes, population norms, or reference values), and varied by the needs of the health care providers in caring for their patients.

The current study reports on a text-based, symptom-monitoring program with patient navigation to assist endometrial, ovarian, and breast patients during treatment. The purpose of the program was to identify patients’ symptoms and needs in a timely manner, before symptoms or problems intensified compromising effective treatment. This paper reports on the feasibility of implementing the program in these patient populations. The a priori goals for program success were that (1) ≥ 85% of patients approached would participate in the monitoring program; and (2) adherence to the surveys during the 12-month period would be ≥ 75% for all cancer types.

## Methods

### Overview

The aims of this program were to (1) monitor patients’ symptoms and needs for up to 12 months during cancer treatment; (2) encourage the use of the patient portal (MyChart) to assist patients in communicating with their health care team and managing their care; and (3) provide navigation services to patients with personal needs that might impede treatment adherence, such as reliable transportation to clinic visits. This program was exempted from human informed consent guidelines by the Institutional Review Board of The Ohio State University as quality improvement. However, all patients had the right to refuse to take part in this clinical quality improvement program.

Patients participated from the Gynecologic Oncology and the Breast Oncology clinics at The Ohio State University Comprehensive Cancer Center (OSUCCC) in Columbus, Ohio. In Gynecologic Oncology, post-operative ovarian and endometrial patients were identified by program staff using the electronic health record (EHR) EPIC. The five participating gynecologic oncologists gave final approval to approach their patients for symptom monitoring. The program was initiated in February 2018 through October 2018. Patients completed symptom surveys using a smartphone, computer, or with staff, and were monitored monthly for 12 months or until the end of active therapy, entry into hospice, or patient or physician request to stop the surveys, whichever came first. English proficiency was not an inclusion criterion. However, we did not have any patients, for whom English was a second language, who could not complete the monthly surveys.

**Fig. 1:**
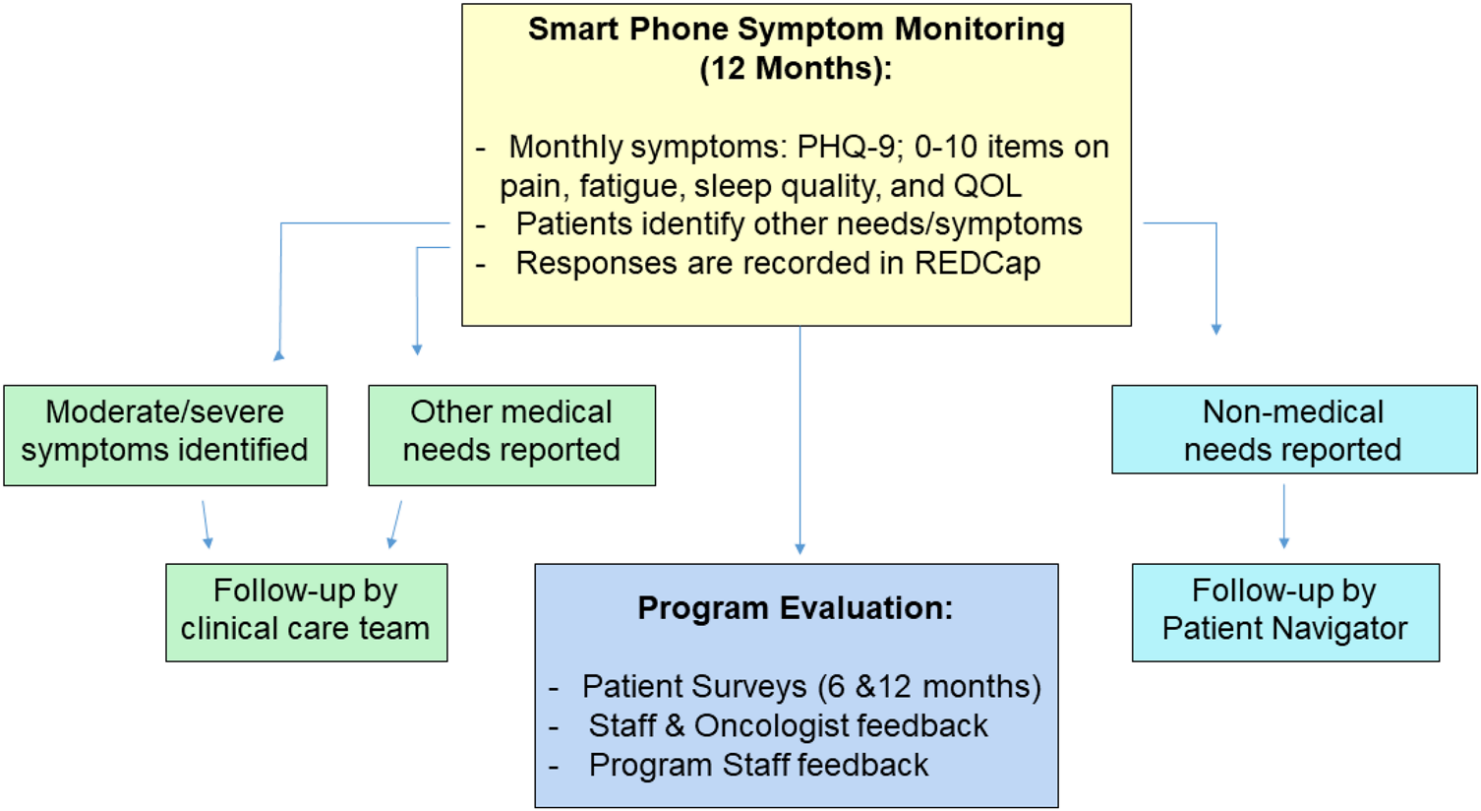
Schema of the symptom-monitoring program

The same procedures were followed in the breast oncology clinic, with the distinction that the three participating oncologists elected to identify patients themselves for the symptom-monitoring program (i.e., the EHR was not used systematically by program staff for patient identification). These patients included those who were either currently undergoing adjuvant therapy or were judged by their health care teams as being able to benefit from additional monitoring or patient navigation. Patients in breast oncology were monitored beginning in December 2018 through June 2019, with follow-up through 12 months or until the end of active therapy or patient or physician request to stop the surveys, whichever came first. Figure 1 provides the schema of the symptom-monitoring program, with details described below.

At program entry, patients were asked to complete brief text-based surveys once a month for up to 12 months. Patients were also encouraged to get an account for the OSU MyChart online patient portal, if they were not currently enrolled, and to sign up to receive text-message or phone call appointment reminders. Program staff assisted interested patients with MyChart and appointment reminder set-ups and provided education in how to use MyChart for their personal care.

Patients completed the first survey in clinic on an iPad using REDCap. Questions were formatted in the same way they would appear in the monthly text surveys to familiarize the patients with the items and the formats. Patients also had the option of completing paper forms or having an interviewer read the questions to them, if they preferred those modes of survey administration. Patient demographic characteristics were obtained/verified by the patients, and included age, race, ethnicity, education, income, employment, and marital status. The patients’ cancer stage was obtained from the OSUCCC cancer registry.

In addition, two subscales from the James Supportive Care Screening [28] measure were given to the patients to complete at program entry, as well as at 6 months (mid-treatment), to identify factors that might impede effective treatment. The 4-item “Health Care Decision-Making and Communication Issues” subscale examines decision-making concerns, problems communicating with the medical team, long-term health care planning, and lack of information about treatment or conditions. The 6-item “Social/Practical Problems” subscale included the patient’s living situation, housing problems, lack of support, financial or insurance problems, transportation problems, and problems obtaining medications. Items on both subscales were scored as 0 = none, 1 = mild, 2 = moderate, 3 = severe. Total subscale scores were calculated by summing the individual subscale items. However, alert values on each individual item were designated as item responses ≥ 2 (i.e., moderate or higher). These high alerts were handled by program patient navigators who telephoned the patients to assess difficulties and provide assistance or referral. The patient navigators worked closely with the social workers and patient care resource managers (PCRM) on each clinical service, as needed. Issues related to clinical concerns were generally handled by the PCRMs, with practical problems handled by the navigators. This patient navigation model was patterned after our past experiences in navigator programs [29]. All patient navigation encounters were documented in REDCap, including the patients’ problems/concerns and services provided.

### Monthly survey administration

Monthly symptom assessments were scheduled and administered through REDCap for up to 12 months. Patients received a survey link via text message or email, and up to three reminders were automated using REDCap if the survey remained incomplete. Survey texts were always sent on a Monday in order for staff to better manage patient alert values on weekdays, if needed. If no surveys were completed in month 1 and/or 2, program staff called patients to ensure receipt of the texts and to adjust their preferred method of contact, if desired. However, after this two-month time period, reminder messages were still delivered monthly, but no additional telephone calls were made to the patient by program staff. For patients who were not comfortable with technology, monthly surveys were conducted by phone or in-person during clinic visits by program staff. Each monthly survey contained the following common items:

### Physical symptom items

Patients completed four items, rated from 0 (low) to 10 (high) during the past 7 days, for pain, fatigue, sleep quality, and overall quality of life [3, 6]. Single-item, numerical linear analogue self-assessment (LASA) scales were used, because they have advantages of being reliable and valid, easily understood by most persons with differing educational backgrounds, and are easier to translate into multiple languages [30, 31]. Patients reporting scores of ≥ 4 for pain or fatigue, or sleep or quality of life < 4 were flagged as patient alert values. These values were pre-determined from the participating clinicians, with in particular, a lower threshold set for symptoms for pain and fatigue, so that these symptoms could be addressed earlier in treatment before symptoms persisted and/or became severe.

### Patient health questionnaire-9 items (PHQ-9)

Gynecologic Oncology patients completed the PHQ-9 for the assessment of depressive and psychological symptoms. This measure is recommended as a valid and reliable screening tool for cancer patients by the American Society of Clinical Oncology [32]. Patients reported on symptoms during the past two weeks, using the following response categories: 0 = not at all, 1 = several days, 2 = more than half the days, and 3 = nearly every day. Scores on the PHQ-9 range from 0 to 27, with scores indicating 0–4 no or minimal depression, 5–9 mild depression, 10–14 moderate depression, 15–19 moderately severe depression, and 20–27 severe depression. Patients were flagged for an alert value if they scored 10 or higher on the PHQ-9 or marked “1” or higher on a single questionnaire item concerning suicidal ideation (i.e., “Thoughts that you would be better off dead or of hurting yourself in some way”). Patients in the breast clinic did not receive the PHQ-9 in their monthly surveys, because this measure was already given routinely as part of their clinical care.

### Other symptoms or needs

Every month, patients were also asked to self-report other major/bothersome symptoms or treatment concerns that they wanted forwarded to their health care team, as well as if they needed assistance with any non-treatment concerns prior to their next clinic visit. Non-treatment concerns (for example, transportation issues, locating supportive services in the community) were forwarded to the patient navigators for follow-up directly with the patient. Treatment or clinic-related issues were sent to each physician’s designated staff person by sending an email or “in-basket” message through the Integrated Healthcare Information System (IHIS) in Epic. The clinic staff person then followed up with the patients regarding their concerns.

### Smartphone provision

Smartphones were used to facilitate communication and to optimize the management of patients’ therapy. Through a partnership with a national wireless company, patients were provided with an iPhone 6 s or 7 if either the patient did not have a smartphone or had a calling plan with limited data or minutes for calling or texting each month. The wireless company provided the phones at zero cost, and program funds paid for phone service for 12 months, including unlimited text messaging and cellular data. At the end of the 12-month program period, the patients were able to keep the iPhones, but had to secure their own phone plan, if desired, or use the phone in venues where wireless internet service was available to complete non-calling or texting functions using the internet.

Program staff helped patients set up the iPhones, including providing basic education on the features of the phone, completed a walkthrough of the MyChart application, signed the patient up for clinic text messages and appointment reminders, if the patients agreed, and installed phone numbers for the oncology clinic, program staff, and supportive services at the cancer center. The iPhone set-up encounters lasted between 30 min to 1 h.

### Symptom reporting to the physicians

Patients’ symptom scores on the monthly surveys were exported from REDCap to a spreadsheet of monthly scores for all measures, and sent to their health care teams using secure email. Alert values for pain, fatigue, poor sleep quality, and quality of life were highlighted on reports, as well as any patient self-reported issues. Program staff also sent the oncology team a direct message in the EHR within 24 h for patients reporting moderate-severe depression on the PHQ-9 (for gynecologic oncology patients) or severe pain (≥ 7 or higher). Oncology teams completed follow-up with the patients and/or placed referrals as necessary, following standard of care procedures. The cut-off scores used for the alert values, the content and presentation of the information put in the spreadsheets, and the process of relaying and responding to the reports were modified over time, based on feedback from the oncology staff and physicians, as well as the patients themselves. For example, patients with spikes in worsening symptoms were highlighted in relation to their past months’ symptom levels to better indicate changes, as well as patients with chronic moderate to severe depressive symptoms who often scored high on the screenings, but were re-verified to ensure that they were receiving follow-up for their depressive symptoms. Individual physicians also could specify what information they wanted reported each month (i.e., only provide high alert values on patients and do not provide any information on patients doing well), and so there was not uniform reporting of information across all physicians after the first several months of monitoring.

### Program evaluation

Both the patients and the participating physicians and lead staff completed structured questionnaires to provide feedback on the content and conduct of the monitoring program. Patients provided feedback as part of the 6-month text-based survey, and provider/lead staff evaluations surveys were emailed to them to complete at 12 months.

## Results

Across the three cancer types, 346 patients were approached to take part in the program with 313 agreeing (90.5%). Program declines by cancer type were 9/79 (11.4%) ovarian; 5/55 (9.1%) endometrial; and 19/213 (9.8%) breast. There were no significant differences in program declines or the reasons for refusing by age, race, ethnicity, or cancer type. The major reasons for not participating were lack of interest (36.4%) or believing the program would not be useful (33.3%).

Demographic and clinical characteristics of the participating patients are provided in Table 1. The breast cancer patients were younger on average than either the ovarian or endometrial patients and were more likely to be married, have higher educational attainment, and be employed. Of note is the wide age range of patients across all three cancer types, with the oldest patients over age 80. The majority of patients were non-Hispanic white, which is indicative of the catchment area of the OSUCCC [33], which is 22% rural and includes the Ohio Appalachian region. Greater than 20% of patients across all cancer types reported incomes below $35,000 per year.

**Table 1:** Demographic and clinical characteristics of the patients in the text-based symptom-monitoring program

	Breast *N* = 193	Ovarian *N* = 70	Endometrial *N* = 50
Characteristics*			
Age [mean (range)]	55.2 (26–82)	62.9 (35–87)	63.1 (43–87)
Race [*n* (%)]			
White	163 (84.5%)	58 (82.9%)	44 (88.0%)
African American or Black	21 (10.9%)	8 (11.4%)	5 (10.0%)
Other	1 (0.5%)	2 (2.9%)	0 (0%)
Mixed/Unknown	8 (4.1%)	2 (2.9%)	1 (2%)
Ethnicity [*n* (%)]			
Not Hispanic/Latina	192 (99.5%)	68 (97.2)	50 (100.0%)
Hispanic/Latina	1 (0.5%)	1 (1.4%)	0 (0%)
Unknown	0 (0%)	1 (1.4%)	0 (0%)
Marital status [*n* (%)]			
Married/living as married	136 (70.5%)	33 (47.1%)	22 (44.0%)
Divorced/Separated	28 (14.5%)	12 (17.1%)	6 (12.0%)
Widowed	11 (5.7%)	6 (8.6%)	2 (4.0%)
Single/Never married	17 (8.8%)	10 (14.3%)	5 (10.0%)
Unknown	1 (0.5%)	9 (12.9%)	15 (30.0%)
Education [*n* (%)]			
≤ High school graduate	35 (18.1%)	16 (22.9%)	17 (34.0%)
Some college/technical school	55 (28.5%)	19 (27.1%)	7 (14.0%)
College graduate	62 (32.1%)	14 (20.0%)	6 (12.0%)
Post-graduate	41 (21.2%)	12 (17.1%)	5 (10.0%)
Unknown	0	9 (12.9%)	15 (30.0%)
Income [*n* (%)]			
< $35,000	44 (22.8%)	15 (23.1%)	11 (20.8%)
$35,000–$49,000	23 (11.9%)	10 (16.1%)	3 (8.6%)
$50,000–$74,999	26 (13.5%)	5 (8.1%)	6 (17.1%)
$75,000–$99,999	26 (13.5%)	6 (9.7%)	2 (5.7%)
> $100,000	44 (22.8%)	12 (19.3%)	3 (8.6%)
Unknown	30 (15.5%)	22 (31.4%)	25 (50.0%)
Employment [*n* (%)]			
Employed	94 (48.7%)	13 (18.6%)	12 (24.0%)
Unemployed	8 (4.1%)	2 (2.9%)	2 (4.0%)
Homemaker	16 (8.3%)	3 (4.3%)	2 (4.0%)
Retired	54 (28.0%)	33 (47.1%)	13 (26.0%)
Disabled	17 (8.8%)	6 (8.5%)	5 (10.0%)
Other	4 (2.1%)	4 (5.7%)	1 (1.0%)
Unknown	0 (0%)	9 (12.9%)	15 (30.0%)
Metropolitan area	145 (75.1%)	50 (71.4%)	27 (54.0%)
Non-Metropolitan area	48 (24.8%)	20 (28.6%)	22 (46.0%)
Cancer stage [*n* (%)]			
0	6 (4.2%)	1 (1.6%)	4 (8.5%)
1	41 (28.5%)	18 (28.1%)	12 (25.5%)
2	45 (31.3%)	16 (25%)	15 (31.9%)
3	25 (17.4%)	21 (32.8%)	12 (25.5%)
4	27 (18.8%)	8 (12.5%)	4 (8.5%)

A summary of key program components is provided in Table 2.

**Table 2:** Characteristics of the text-based symptom-monitoring program by cancer type

	Ovarian cancer (*N* = 70) [*n* (%)]	Endometrial cancer (*N* = 50) [*n* (%)]	Breast cancer (*N* = 193) [*n* (%)]
Already enrolled in MyChart	50 (71.4%)	27 (54.0%)	155 (78.8%)
MyChart enrollment refusals among those not already enrolled*	10/20 (50.0%)	11/23 (47.8%)	10/28 (35.7%)
Provided with an iPhone	10 (14.3%)	4 (8.0%)	28 (14.2%)
Adherence to monthly surveys [% (range)]	77.5% (67%-88%)	75.5% (71–86%)	73.5% (64–83%)
Staff administered monthly surveys	7 (10.0%)	4 (8.0%)	10 (5.1%)
Patient withdraws	5 (7.1%)	9 (18.0%)	13 (6.7%)
Patient deaths	17 (24.2%)	2 (4.0%)	11 (5.7%)
PHQ-9 scores ≥ 10 and/or with suicidal ideation	24 (34.3%)	15 (30.0%)	0 (0.0%)**
Received navigation services	9 (12.9%)	7 (14.0%)	53 (27.4%)

*This is presented as the number of patients refusing to enroll divided by the number of patients not enrolled in MyChart at the start of the symptom-monitoring program

**The PHQ-9 was not administered in the breast clinic as part of this program

### MyChart

At program entry, 74.1% of all patients were already enrolled in MyChart. Patients not enrolled were asked to enroll, with refusals ranging between 37 and 50% of the non-enrolled patients. Reasons for refusing were that they did not have reliable access to the internet or computers, preferred to call or talk to health professionals in person, or simply were not interested in using MyChart. Approximately 55% of patients over age 65 refused enrollment in MyChart, primarily among the ovarian and endometrial patient groups.

### iPhone provision

iPhones were provided to 42 (13.4%) patients across all cancer types. Demographic characteristics of the patients who received iPhones were compared with those who already had a smartphone, with patients receiving iPhones having incomes below $50,000/year (*p* = 0.03) and an educational level of high school or less (p < 0.0001). Program staff had few difficulties training patients to operate the phones correctly or in patients’ adherence to completing surveys after receiving the iPhones. Phone service charges for patients receiving iPhones averaged approximately $40 per month or $500 per person for the 12-month period.

### Survey adherence and mode of administration

**Fig. 2:**
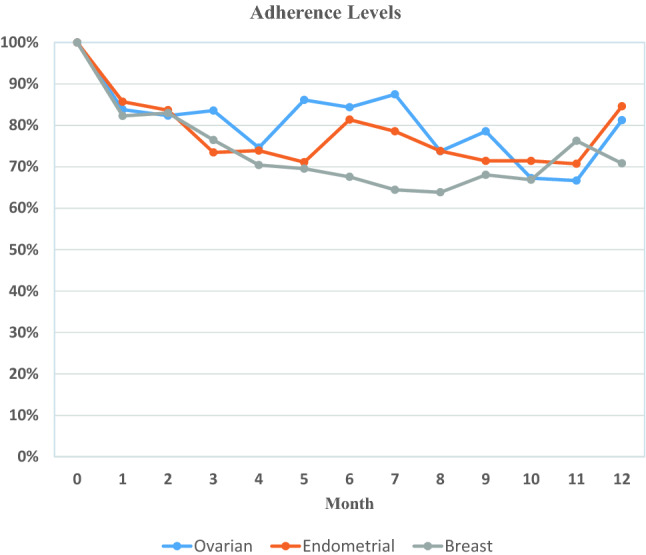
Adherence to completing the monthly surveys by cancer type

Figure 2 shows the proportion of patients who completed the surveys at each time point. Adherence averaged between 75 and 77% overall with responses varying by cancer type, as well as the month of assessment. Patients were censored at the time of their formal withdrawal from the program or death, so that the monthly percentages only include active patients who completed the surveys at each time point. At month 5, there began a decline in monthly survey adherence, particularly among the breast patients, coinciding with the completion of chemotherapy/radiation treatments, as patients completed active therapy. However, unless patients asked to be formally withdrawn from the program, they continued to receive the monthly surveys. Formal patient withdraws were highest among ovarian and endometrial cancer patients due to death, disease progression, or entering hospice during the monitoring period (Table 2).

Of note is that there was no difference in response rates to the monthly surveys by age or in older patients’ abilities to use or be trained to use the iPhone to complete the surveys. Only a small number of patients (*n* = 21, 6.7%) preferred to have one or more of the monthly surveys administered by program staff via telephone or in-person. The majority of these patients were > age 70 and/or without reliable internet access, which is not uncommon in rural areas and the Appalachian region in Ohio. No patients completed the monthly surveys on paper forms after baseline.

### Symptom alert values

**Fig. 3:**
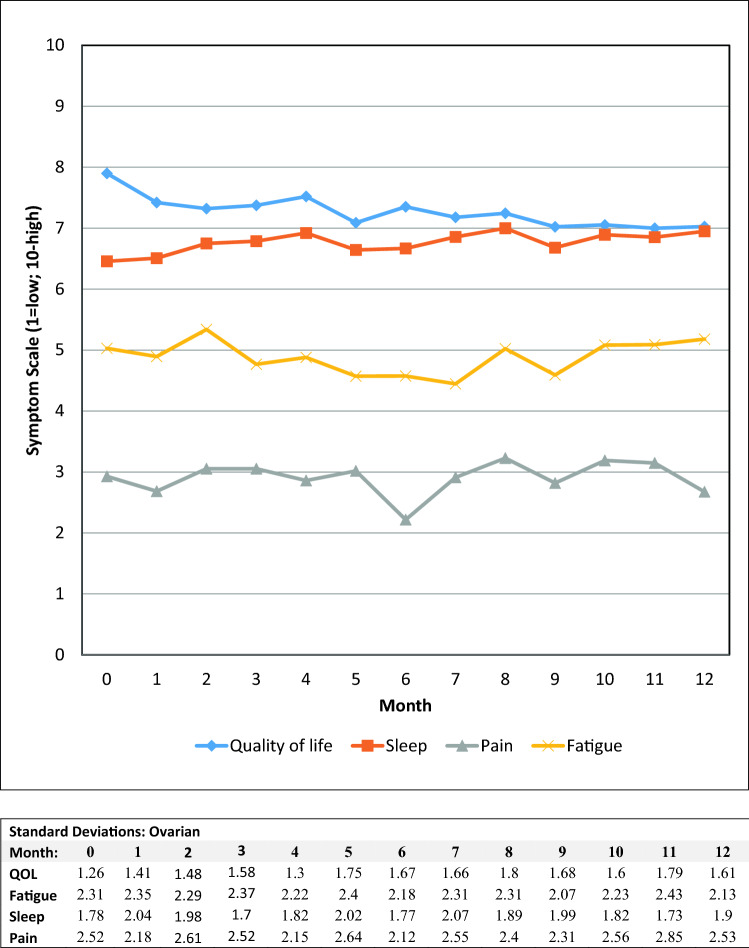
Mean symptom values by month for ovarian patients

**Fig. 4:**
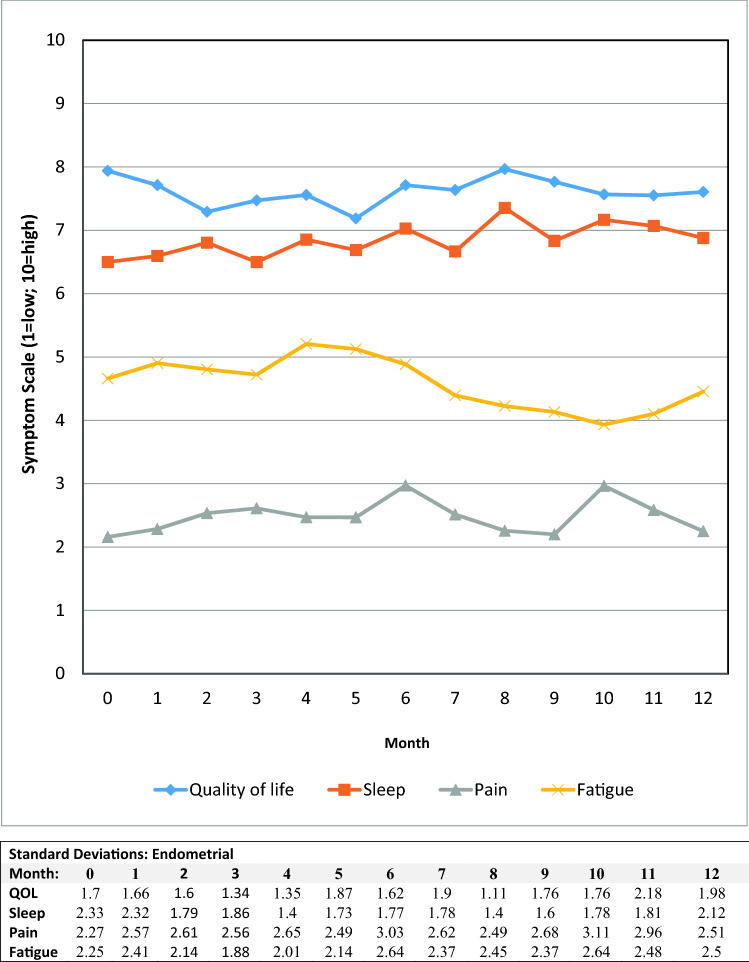
Mean symptom values by month for endometrial patients

**Fig. 5:**
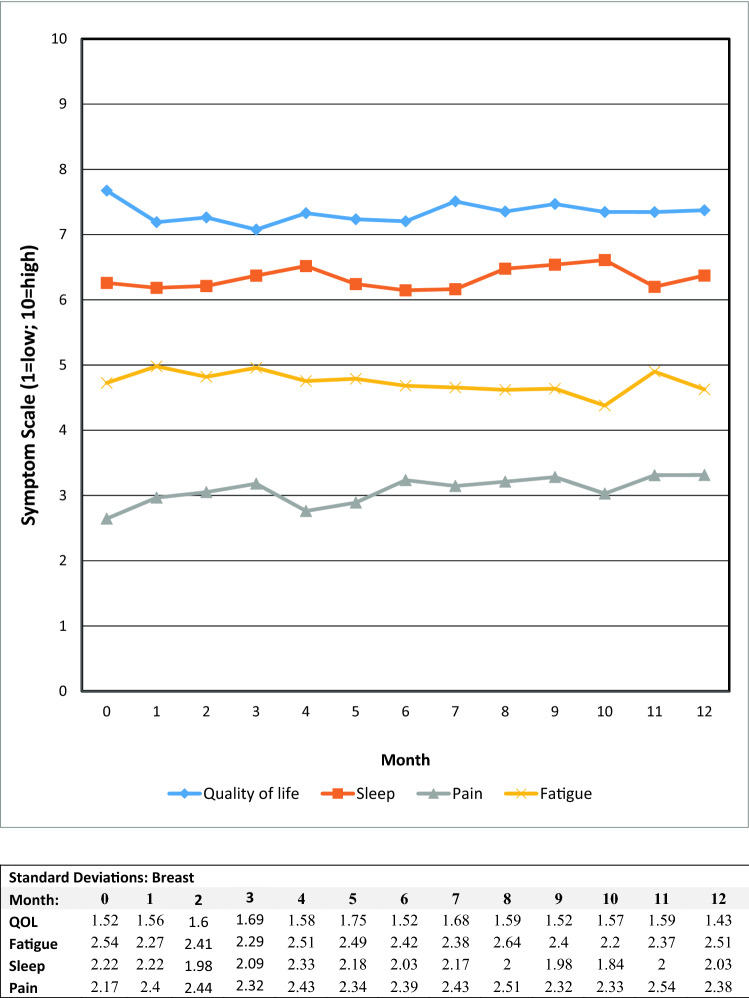
Mean symptom values by month for breast patients

Alert values for PHQ-9 scores ≥ 10 occurred in roughly one-third of the gynecologic oncology patients, with 3% expressing suicidal ideation. The majority of these patients were already receiving behavioral health services, with those not under care referred to behavioral health services in their areas. Graphs of the mean scores for the fatigue, sleep quality, pain, and quality of life 0–10 items are presented in Figs. 3, 4, and 5. The major persistent symptoms over the 12-month period, across all cancer types, were moderate levels (i.e., 4–7) of fatigue and poorer sleep quality. Pain was generally well controlled for all patient groups, and overall quality of life averaged between 6 and 8 for all patient groups.

### Patient navigation

Navigation was used by approximately 13% of the ovarian, 14% of the endometrial cancer patients, and 27% of breast cancer patients. Navigators contacted the patients based on their responses to the monthly text-based surveys, as well as their responses to the two subscales of the James Supportive Care Questionnaire at baseline and month 6. Types of services for which patients needed assistance included transportation to and from clinic visits, information about cancer support groups and supportive services, cancer-related information, treatment questions, financial or insurance concerns, such as assistance with paying for medications or monthly bills, and dealing with insurance issues. Questions about treatment, medications, and insurance were forwarded to the nurse PCRMs in each clinic, after the navigator had talked with the patients to better understand their needs. Assistance with transportation, information about supportive services, or social programs to assist with monthly bills or housing were handled by the navigators. The average numbers of encounters the navigators had in working with each patient was between 2 and 3, with the majority of these encounters handled by telephone rather than in-person. Reported problems decreased between program entry and 6 months, as patient problems and needs were addressed (Table 3).

**Table 3:** James supportive care subscale responses at program entry and 6 months

	Breast		Endometrial		Ovarian	
	Baseline (*N* = 193)	6 Months (*n*-119)	Baseline (*N* = 47)	6 Months (*N* = 30)	Baseline (*N* = 66)	6 months (*N* = 45)
Health care decision-making concerns						
None	111 (57.5%)	84 (70.6%)	31 (66.0%)	22 (73.3%)	40 (60.6%)	35 (79.6%)
Mild	48 (24.9%)	20 (16.8%)	8 (17.0%)	5 (16.7%)	17 (25.8%)	5 (11.4%)
Moderate	30 (15.5%)	12 (10.1%)	7 (14.9%)	3 (10%)	8 (12.1%)	4 (9.1%)
Severe	4 (2.1%)	3 (2.5%)	1 (2.1%)	0	1 (1.5%)	0
Problems communicating with medical team						
None	170 (88.1%)	109 (91.6%)	45 (95.7%)	26 (86.7%)	55 (84.6%)	43 (95.6%)
Mild	15 (7.8%)	4 (3.4%)	1 (2.1%)	3 (10%)	9 (13.9%)	2 (4.4%)
Moderate	6 (3.1%)	6 (5.0%)	1 (2.1%)	1 (3.3%)	0	0
Severe	2 (1.0%)	0	0	0	1 (1.5%)	0
Long-term health care planning concerns						
None	124 (64.3%)	77 (64.7%)	29 (64.4%)	20 (66.7%)	41 (63.1%)	35 (77.8%)
Mild	36 (18.7%)	27 (22.7%)	12 (26.7%)	8 (26.7%)	17 (26.2%)	7 (15.6%)
Moderate	29 (15.0%)	14 (11.8%)	4 (8.9%)	2 (6.7%)	6 (9.2%)	3 (6.7%)
Severe	4 (2.1%)	1 (0.8%)	0	0	1 (1.5%)	0
Lack of information about their treatment or condition						
None	171 (88.6%)	101 (84.9%)	40 (90.9%)	28 (93.3%)	61 (92.4%)	40 (88.9%)
Mild	17 (8.8%)	13 (10.9%)	3 (6.8%)	1 (3.3%)	5 (7.6%)	4 (8.9%)
Moderate	5 (2.6%)	5 (4.2%)	1 (2.3%)	0	0	1 (2.2%)
Severe	0	0	0	1 (3.3%)	0	0
Concerns about their living situation						
None	154 (80.2%)	104 (87.4%)	40 (85.1%)	28 (93.3%)	55 (83.3%)	41 (93.2%)
Mild	22 (11.5%)	10 (8.4%)	4 (8.5%)	1 (3.3%)	9 (13.6%)	1 (2.3%)
Moderate	11 (5.7%)	3 (2.5%)	3 (6.4%)	1 (3.3%)	2 (3.0%)	2 (4.5%)
Severe	5 (2.6%)	2 (1.7%)	0	0	0	0
Housing problems						
None	164 (85.4%)	111 (93.3%)	43 (93.5%)	29 (96.7%)	61 (92.4%)	41 (93.2%)
Mild	18 (9.4%)	2 (1.7%)	2 (4.4%)	1 (3.3%)	5 (7.6%)	1 (2.3%)
Moderate	6 (3.1%)	4 (3.4%)	1 (2.2%)	0	0	1 (2.3%)
Severe	4 (2.1%)	2 (1.7%)	0	0	0	1 (2.3%)
Lack of support						
None	160 (83.3%)	103 (86.6%)	44 (95.7%)	26 (86.7%)	61 (93.9%)	40 (90.9%)
Mild	25 (13.0%)	12 (10.1%)	2 (4.4%)	3 (10%)	3 (4.6%)	4 (9.1%)
Moderate	5 (2.6%)	3 (2.5%)	0	1 (3.3%)	1 (1.5%)	0
Severe	2 (1.0%)	1 (0.8%)	0	0	0	0
Financial of insurance problems						
None	116 (60.4%)	75 (63.0%)	30 (63.8%)	16 (53.3%)	43 (66.2%)	32 (72.7%)
Mild	43 (22.4%)	29 (24.4%)	11 (23.4%)	11 (36.7%)	11 (16.9%)	6 (13.6%)
Moderate	26 (13.5%)	10 (8.4%)	5 (10.6%)	3 (10%)	9 (13.8%)	5 (11.4%)
Severe	7 (3.7%)	5 (4.2%)	1 (2.1%)	0	2 (3.1%)	1 (2.3%)
Transportation problems						
None	169 (88%)	112 (94.1%)	40 (85.1%)	28 (93.3%)	60 (90.9%)	40 (90.9%)
Mild	17 (8.9%)	5 (4.2%)	6 (12.8%)	2 (6.7%)	2 (3.0%)	4 (9.9%)
Moderate	5 (2.6%)	1 (0.8%)	1 (2.1%)	0	3 (4.6%)	0
Severe	1 (0.5%)	1 (0.8%)	0	0	1 (1.5%)	0
Problems obtaining medications						
None	169 (88.0%)	104 (87.4%)	46 (97.9%)	30 (100%)	61 (93.9%)	42 (95.4%)
Mild	17 (8.9%)	7 (5.9%)	1 (2.1%)	0	2 (3.1%)	1 (2.3%)
Moderate	5 (2.6%)	6 (5.0%)	0	0	2 (3.1%)	1 (2.3%)
Severe	1 (0.5%)	2 (1.7%)	0	0	0	0

### Patient evaluation

Formal quantitative evaluations of the delivery and value of the program were conducted with the patients at month 6 (all cancer types) (Table 4). Between 97.5 and 100% found it easy to complete the surveys on their phone/computer, and 71–77% found the program to be useful to themselves and their health care teams. Approximately 81% of the breast and 77.6% of the gynecologic oncology patients believed the monthly symptom questions helped them communicate better with their health care team. Approximately 86% of the endometrial and ovarian, and 92% of the breast cancer patients also believed other patients would benefit from the program during their treatment. Patients not finding the monthly surveys useful primarily commented that the questions were too redundant with assessments during treatment visits, that they were already cognizant of their symptom levels, and were not hesitant to talk to their health care provider regarding their concerns during clinic visits.

**Table 4:** Evaluation of the monitoring program by the patients at 6 months post-enrollment

	Breast (*N* = 119) (%)	Endometrial and ovarian (*N* = 82) (%)
*(% responding “All or most of the time”)*		
Monthly surveys are easy to complete on my phone or computer	100	97.5
Able to recognize that the monthly text or email messages are coming from the OSU clinic	98.3	98.7
Being asked about my symptoms each month was useful to me and my health care team	71.2	76.9
Liked being monitored for symptoms each month	72.0	75.6
Liked being asked each month if I needed assistance with anything prior to my next clinic visit	76.3	76.8
*(% responding “Strongly Agree or Agree”)*		
Symptom questions helped me communicate better with my oncologist and the staff	81.0	77.6
Think my oncologist/staff reviewed my answers on the surveys each month	86.4	80.6
Think my oncologist made recommendations for my care based on some of my answers on these monthly surveys	79.5	76.1
Think other patients would benefit from receiving these monthly surveys while they are receiving treatment	92.4	86.5

Patients were also asked at 6 months about the frequency of receiving the surveys. 92% of ovarian and endometrial, and 81% of breast patients believed that receiving the surveys once a month was “just about right.” The remainder suggested completing surveys at 6-week to 3-month intervals, depending on the stage of a patient’s treatment and when they were scheduled to be seen in clinic.

### Oncologist/lead staff evaluation

The oncologists and lead staff provided feedback on the symptom-monitoring program through an emailed survey, with a follow-up interview or further email correspondence used with some providers to better understand suggestions/concerns for program improvement. In both gynecologic oncology and breast oncology, we had a lead oncologist or “clinic champion” who helped design the program for use with the target populations, and bring other oncologists onboard to participate in the program. Components considered to be the most effective were patients completing the surveys on smartphones/electronic devices, encouraging patients to use MyChart, and the ability for patients to be linked to a patient navigator for assistance. Several oncologists/staff were surprised to learn that some patients were more forthcoming on the surveys than when they talked to them in clinic, which opened up better communication with their patients. However, an unintended consequence was that a small number of patients (< 10) waited until their monthly survey was due to report severe symptoms to their oncologist, instead of reporting concerns to their health care team when they occurred. This sometimes led to a delay in treating symptoms.

Suggestions for improving the program were to find more succinct ways to report patient alert values to the health care team, including using patient graphs of symptoms over time; only reporting on patients each month who had alert values; focusing primarily on severe versus moderate symptoms; timing some assessments to be completed a week prior to the patients’ next clinic visit instead of only at monthly intervals; allowing oncologists to tailor the timing and content of the symptom monitoring to match specific patient’s needs; and including a report of the services provided by the patient navigator in the monthly report. In addition, several oncologists were uncertain of the value of continuing the monthly surveys after their patients had completed active therapy, given that patients were only being scheduled to come back to clinic at 3- or 6-month intervals for follow-up visits, and patients should be transitioning back to primary care or their routine health care providers.

## Discussion

This paper reported on the feasibility of implementing text-based symptom monitoring with patient navigation to assist ovarian, endometrial, and breast cancer patients undergoing treatment. A major focus was on developing a symptom-monitoring system that could be utilized by most patients, and did not perpetuate biases against patients who lacked electronic devices. Unique aspects of this program included being able to provide smartphones and training to patients without these devices, as well as institute alert values to trigger patient navigators to triage patients’ clinical and non-clinical care needs. Our focus was primarily on larger health systems or academic medical centers that may have resources either through research grant funds or other sources to support these programs. We also sought to utilize or build on existing resources to offset costs of this program. For example, REDCap is available to many academic health centers in the U.S., and can support these types of monitoring program economically.

Successes of this program were that greater than 90% of patients in all three clinics elected to participate in this voluntary activity and complete the text-based surveys for up to 12 months. Adherence to the monthly surveys averaged to approximately 75%, but adherence was lowest among breast patients after 5 months, coinciding in part, with the completion of active therapy. In general, the majority of patients reported value in completing the monthly surveys and having another means to communicate with their health care team. The oncologists and staff in the participating clinics provided critical feedback. They found merit in being able to monitor patients remotely between clinic visits, although the frequency and the timing of the patient assessments, instructions given to patients to contact their health care team directly with severe symptoms or concerns, and the presentation of the survey results back to the health care team will need further streamlining. In addition, “real-time” symptom reporting to the health care team was requested, and the ability to focus on select patients with customized monitoring was believed to be an important use of this technology moving forward. The provision of cell phones to patients, as well as providing navigation services, went smoothly with no difficulties. A limitation of this program, however, was that since this was a pilot quality improvement program and not a randomized intervention study, there were no control groups for comparison purposes. In addition, although we made headway in training older patients to use smartphones or other electric devices to complete the monthly surveys, we still had greater numbers of older patients who preferred to complete these assessments by telephone administration and/or to refuse to enroll in MyChart. These results are similar to those reported by other investigators [21, 23, 27].

A recent review of mobile health interventions/programs found a positive impact among application users in the area of improved symptom control, and determined that changing the patterns of communication between patients and providers is one of the most beneficial aspects of mobile health [25]. Patients in our text-based program also reported similar benefits with more than 70% of patients indicating that completing the symptom surveys helped them communicate better with their providers. In addition, greater than 85% thought that other patients would benefit from this type of symptom monitoring during treatment.

Our original intent was to develop a system that could utilize MyChart to collect patients’ symptoms and needs over time. However, a major drawback of using MyChart is that it lacks flexibility in being able to more quickly add or modify questionnaire items, unlike REDCap. In addition, lower enrollment in MyChart among older adult patients, who constitute the majority of cancer patients, and/or those without electronic devices, again excludes patients from such monitoring and perpetuates health disparities. This is particularly problematic in the state of Ohio, given the large rural and Appalachian populations with sometimes unreliable internet service. Thus, we elected to use REDCap as our mode of survey delivery and data capture. This system worked well for our program purposes, and was very efficient and easy to manage. We will continue to refine this program to discern who might benefit the most from this type of monitoring, how best to meet the needs of the oncologists and staff in treating their patients, and explore options to integrate these data into the EHR, if desired by the health care teams, or use MyChart for some program components.

The value of this and other similar programs will be determined by whether they result in cost savings in terms of fewer hospitalizations, emergency department visits, having patients with better mental health and social support, or assist patients to solve personal/economic barriers to treatment through the use of patient navigators. Not all health care systems can access all of these program components, but routine symptom monitoring using a smart phone or computer/website may be accessible to many. Monitoring can be done in a variety of different ways when patients are not in clinic. This program was just one of the ways patient’ symptoms could be assessed in “real time” using a common technology to address patient needs while undergoing therapy.

## Conclusion

This study established the feasibility of implementing a text-based, symptom management program with navigation support for cancer patients undergoing treatment. Future reports will examine the outcomes of this program on clinic flow and metrics (emergency department visits, hospitalizations), as well as more in-depth analyses of the impacts on patient quality of life.
